# a-Si:H/SiNW shell/core for SiNW solar cell applications

**DOI:** 10.1186/1556-276X-8-466

**Published:** 2013-11-06

**Authors:** Eman Sad Ashour, Mohamad Yusof Bin Sulaiman, Mohd Hafidz Ruslan, Kamaruzzaman Sopian

**Affiliations:** 1 Department of Physics, Faculty of Science and Technology, Universiti Kebangsaan Malaysia (UKM), Bangi 43600, Malaysia; 2 Solar Energy Research Institute (SERI), Universiti Kebangsaan Malaysia (UKM), Bangi 43600, Malaysia

**Keywords:** Silicon nanowire, Solar cells, a-Si:H, Antireflection coating, Metal-assisted wet chemical etching, PECVD

## Abstract

Vertically aligned silicon nanowires have been synthesized by the chemical etching of silicon wafers. The influence of a hydrogenated amorphous silicon (a-Si:H) layer (shell) on top of a silicon nanowire (SiNW) solar cell has been investigated. The optical properties of a-Si:H/SiNWs and SiNWs are examined in terms of optical reflection and absorption properties. In the presence of the a-Si:H shell, 5.2% reflection ratio in the spectral range (250 to 1,000 nm) is achieved with a superior absorption property with an average over 87% of the incident light. In addition, the characteristics of the solar cell have been significantly improved, which exhibits higher open-circuit voltage, short-circuit current, and efficiency by more than 15%, 12%, and 37%, respectively, compared with planar SiNW solar cells. Based on the current–voltage measurements and morphology results, we show that the a-Si:H shell can passivate the defects generated by wet etching processes.

## Background

Silicon nanowires (SiNWs) attract significant attention because of their potential applications in many fields like sensors, transistors, lithium batteries, diodes, and photovoltaics [1-5]. Particularly, they can be applied on silicon solar cells as an antireflection coating, due to low average reflectance values [6,7]. Several synthesis methods have been used to fabricate SiNWs including chemical vapor deposition [8], laser ablation [9], thermal evaporation, and solution methods [10-12]. Among these synthesis methods, wet chemical etching has been frequently used to prepare SiNWs. Metal-assisted wet chemical etching is advantageous for achieving SiNWs with controlled diameter, length, spacing, and density, avoiding expensive and low-throughput usual lithographic processes [13].

Recently, it has been shown that a silicon nanowire antireflection coating (ARC) prepared by metal-assisted wet chemical etching is a near-perfect antireflection coating [14]. The superior antireflection property of the nanowire surface is attributed to three reasons: huge surface area of SiNWs, rough surface morphology which leads to strong light scattering as well as absorption, and graded refractive index profile between air and SiNWs that closely implies a multilayer antireflection coating [6,14,15]. Some other properties of SiNWs, for example, crystal ordination, good doping level, and excellent uniformity, imply appropriate utilization of SiNWs in silicon solar cells.

Despite all these features, the maximum efficiency of planar solar cells using SiNW ARC does not exceed 10%. This low efficiency is attributed to many factors. One of the most important is the surface recombination velocity which strongly increases when using SiNW ARC, due to the large surface area [16,17]. It is necessary, therefore, to passivate the SiNW surface, minimizing the surface states [18].

Among all materials used to passivate planar silicon solar cells, hydrogenated amorphous silicon (a-Si:H) has attracted much attention since Sanyo's first declaration of the heterojunction with intrinsic thin layer (HIT) solar cell design [19]. From that time, HIT solar cell efficiency exceeds 22%, and the surface passivation capability of a-Si:H was intensively studied [19,20]. Finding that interstitial a-Si:H is the main cause of reduction of the surface state density results in high-quality passivation of the silicon surface [21,22]. Additionally, a thin layer of a-Si:H was proved to passivate all types of silicon substrates with the entire doping levels. Being deposited at temperatures below 250°C was a merit that leads to a decrease in the thermal budget of solar cell production processes. In this respect, a-Si:H is expected to be a good passivation choice for Si nanostructure solar cells. Crozier et al. [16] demonstrated that *in situ* amorphous Si/SiNW surface recombination decayed just about 2 orders of magnitude compared with SiNWs alone. The surface passivation capability of amorphous silicon was proved by the increase of lifetime and carrier diffusion length. However, this passivation effect was not investigated on the SiNW solar cell performance. In a previous study [16], SiNWs were synthesized using the VLS process which was a bottom-up synthesis approach. Indeed, those SiNWs differ from SiNWs synthesized by metal-assisted wet chemical etching (top-down approach), especially in the defect type and quantity, SiNW density, as well as doping mechanism [23].

In this work, for the first time, the fabrication of an a-Si:H/vertically aligned SiNW (shell/core) solar cell was proposed. The SiNW arrays were fabricated by metal-assisted wet chemical etching of silicon substrates, whereas the a-Si:H shell was deposited by plasma-enhanced chemical vapor deposition (PECVD). The structural, optical, and electrical properties of the a-Si:H/SiNW solar cell were all analyzed.

## Methods

The growth of aligned SiNW arrays was carried out on p-type (100) silicon (0 to 1 Ω cm) wafers. The etching was carried out in a Teflon beaker containing a HF/AgNO_3_ solution, varying etching parameters like concentration, temperature as well as etching time. Prior to the etching, the samples were sequentially cleaned with acetone, ethanol, and de-ionized water for 5 min each followed by cleaning with a boiling piranha solution (H_2_SO_4_/H_2_O_2_ = 3:1 by volume, for 60 min) to remove any organic containment. The samples were then rinsed thoroughly with de-ionized water followed by dipping in 10% HF solution to remove any surface oxides. The cleaned silicon wafers were then immersed in the etching solution HF/AgNO_3_ (5.25:0.02 M). After the etching processes, the tree-like silver pattern wrapping the silicon samples was detached using a NH_3_OH/H_2_O_2_ (3:1) solution. Finally, the samples were rinsed with de-ionized water and air-dried.

A conventional diffusion procedure was carried out to fabricate the SiNW solar cell. The n + emitter was formed by phosphorus diffusion using a POCl_3_ liquid source at 850°C for 20 min. The phosphosilicate glass (PSG) that formed during diffusion was removed by dipping the samples in 5% HF for 2 min.

Hydrogenated amorphous silicon was deposited on the surface of SiNWs by PECVD. The deposition occurred under the following conditions: a power of 100 W, a temperature of 150°C, a pressure of 1 Torr, and a SiH_4_ gas flow of 26 sccm.

The Al back contact with 2,000-nm thickness was formed using an electron beam evaporator (Edwards Auto 306 Turbo, Sanborn, NY, USA). In order to form the back surface field (BSF), alloying of Al and Si was carried out at 900°C. The front metal contacts were made by Ag deposition (180 nm) through a metal mask using the same e-beam evaporator followed by contact sintering in forming gas at 450°C. Finally, 1 × 1 cm^2^ solar cells were diced for electrical characterization.

The morphology of the samples was examined using a field emission scanning electron microscope (FESEM; Carl Zeiss Supra 55VP, Oberkochen, Germany). The structure and chemical composition of the samples were investigated by Fourier transform infrared spectroscopy (FTIR). Reflection (R) spectra were obtained using a Shimadzu UV-3600 spectrophotometer (Kyoto, Japan). The *J*-*V* characteristics of the devices were measured with Keithley 237 SMU (Cleveland, OH, USA) under illumination at 100 mW/cm^2^ from a solar simulator with an AM 1.5G filter.

## Results and discussion

**Figure 1:**
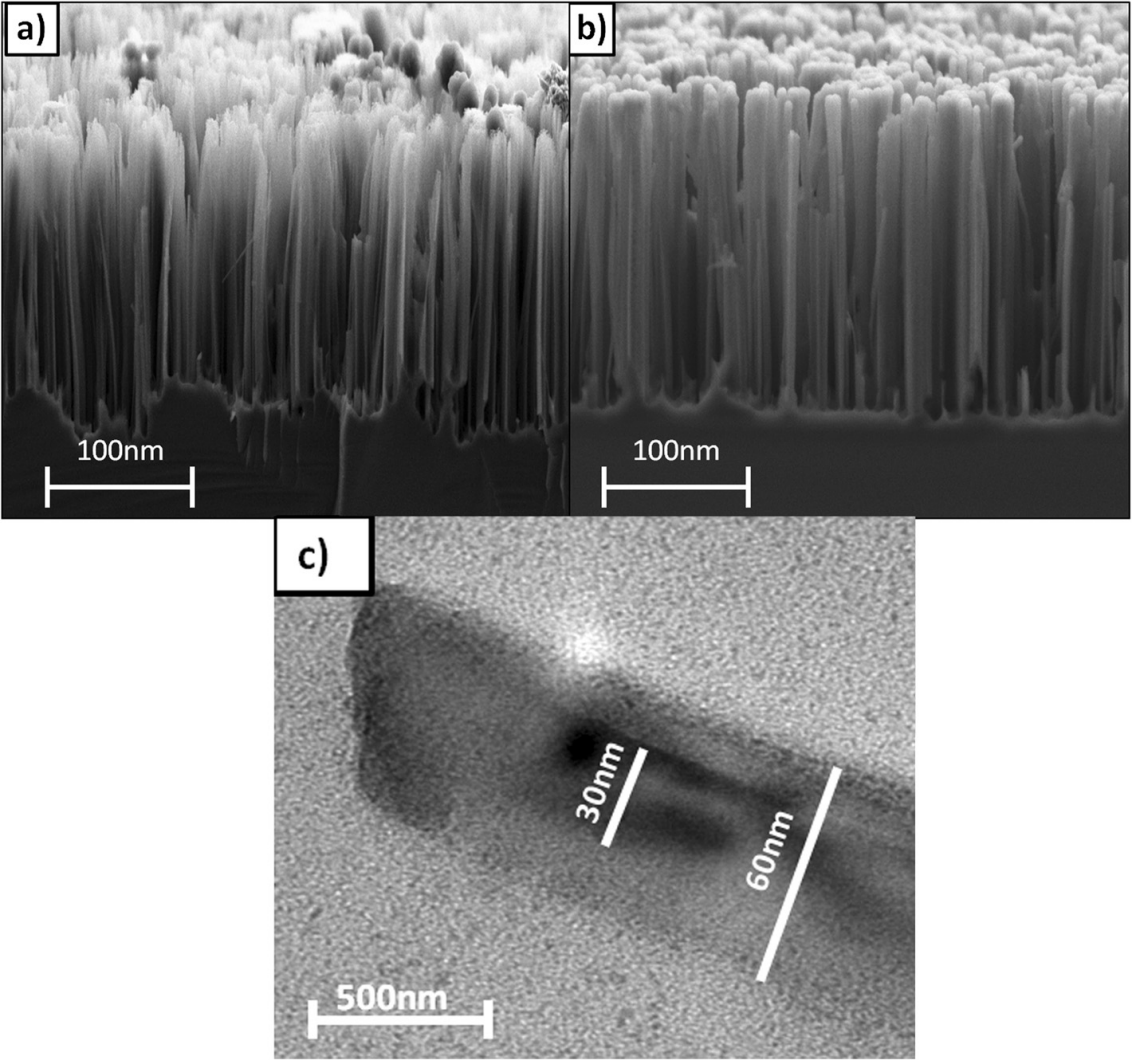
**FESEM and TEM images of the SiNWs and a-Si:H/SiNWs. (a**, **b)** FESEM images of SiNWs and a-Si:H/SiNWs, respectively. **(c)** TEM image of the a-Si:H shell over the SiNWs.

The cross-sectional views of the SiNWs and a-Si:H/SiNWs were investigated using FESEM as shown in Figure 1. Vertically aligned SiNWs were uniformly distributed over the whole area of the silicon surface with 3-μm length. While comparing SiNW and a-Si:H/SiNW structures, it was observed that the deposited a-Si:H filled the SiNW surface with a thin shell. The transmission electron microscopy (TEM) image in Figure 1c indicates that the thickness of the deposited a-Si:H is around 30 nm. Additionally, the TEM image presents the homogenous and uniform a-Si:H shell over the SiNWs.

**Figure 2:**
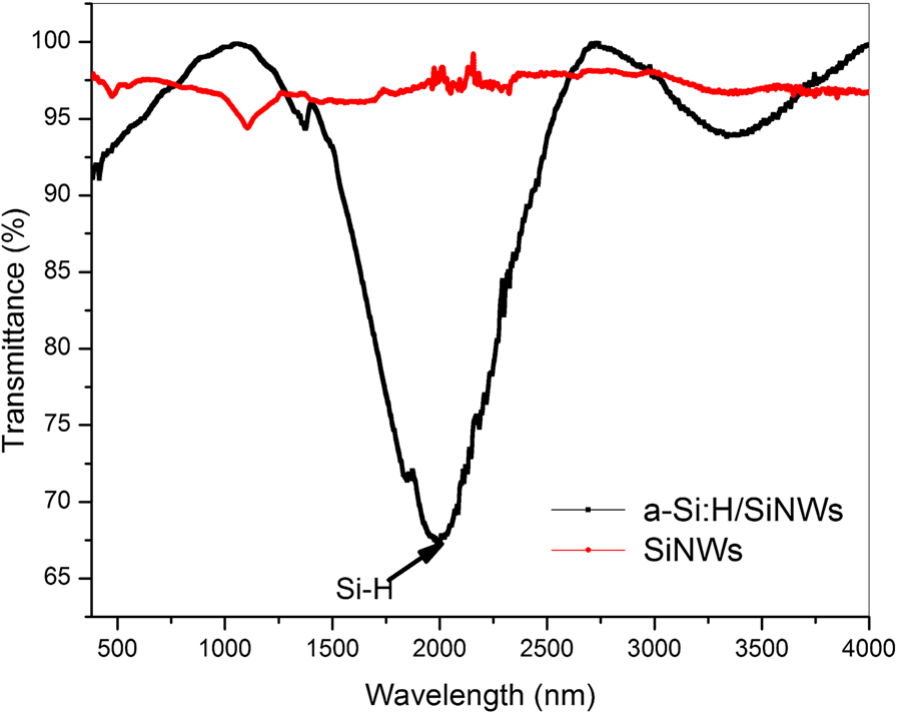
Transmittance spectra of planar SiNWs and thin a-Si:H shell.

Figure 2 highlights the FTIR transmittance spectra of both planar SiNWs and thin a-Si:H shell deposited on the SiNW core by PECVD for 3 min. While investigating the planar SiNW FTIR spectrum, the main peak appeared at 1,105 cm^-1^; it is mostly the signature of the asymmetrical stretching of the Si-O-Si bond, and relying on previous works, it is mainly related to the silicon substrate [24]. For a-Si:H/SiNWs, a broad band around 2,000.22 cm^-1^ emerged normally owing to the stretching mode of the Si-H bond [25]. The full width at half maximum (FWHM) of the Si-H peak was in the same range as that of the reference a-Si:H deposited by PECVD under the same conditions. Since the a-Si:H shell was not annealed after deposition, no narrowing of the stretch peak was observed [26]. The high FWHM of the Si-H peak is mostly related to the high content of hydrogen of the deposited a-Si:H [24]. The high hydrogen content of the a-Si:H shell is suggested to have a good-quality passivation effect [27]. In summary, the FTIR spectrum confirms the deposition of the a-Si:H over SiNWs with appropriate features.

**Figure 3:**
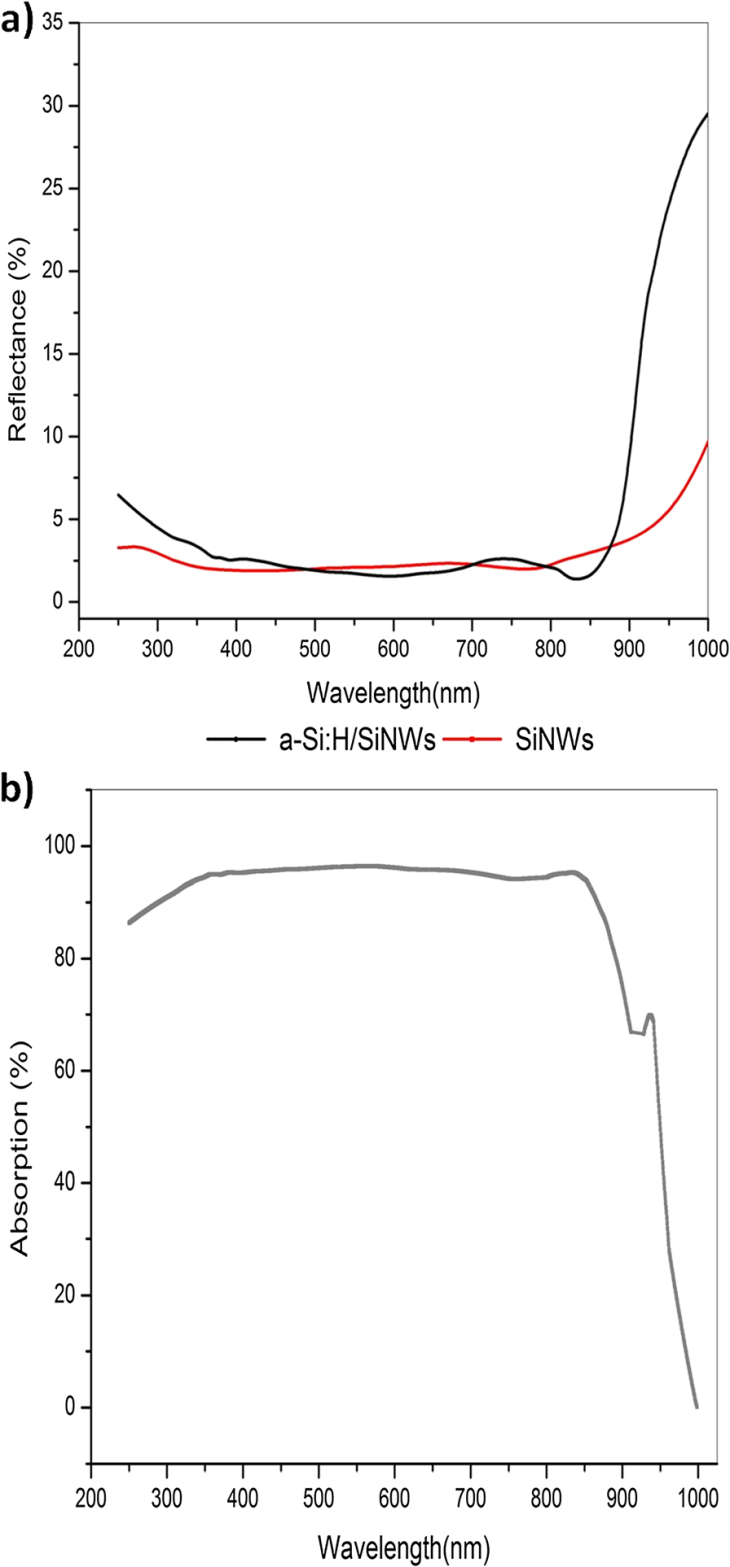
Reflection spectrum of a-Si:H/SiNWs and SiNWs (a) and absorption spectrum from reflection and transmission results (b).

Figure 3 presents the reflection spectrum of a-Si:H/SiNWs and SiNWs. a-Si:H/SiNWs had suppressed the reflection to low values at incident light wavelength ranges from 250 to 1,000 nm. As noted, the combination of a-Si:H shell over SiNW core reduces the average reflectance as low as 5.2%. Relying on previous studies, the low reflection of a-Si:H/SiNWs is mainly caused by the graded refractive index of the SiNW core [28]. Moreover, the filling ratio between the SiNWs and substrate surface plays a vital role in reducing the reflection of the core/shell structures. While studying the a-Si:H thickness effect on the filling ratio, 30 nm was found to be the optimum thickness with respect to both the filling ratio and hence the light reflection [29].

Going back to earlier works, a-Si:H thin films reflect more than 45% of the incident light [30]. Thus, it is expected that the a-Si:H/SiNW structure will be a sufficient antireflection coating combining amorphous and crystalline silicon features.

The absorption spectrum that was extracted from the measured reflection and transmission results is shown in Figure 3. It is noticeable that a-Si:H/SiNWs show a superior absorption property with an average over 87% of the incident light. Note here that the recent simulated results predicted the absorption to be around 60% to 75% [29] for 1-μm thickness. Using SiNWs with 3-μm lengths in this work could be the cause of such increment. As well known, SiNWs reflect less light while increasing their thickness [18].

Another inspiring feature of the a-Si:H/SiNW absorption spectrum is the shifting of the absorbed edge to near-infrared wavelengths. This shifting confirms the dual absorption function of both a-Si:H and SiNWs. Basically, each of them absorbed the wavelengths of the light which match to their energies. Comparing the absorption edges of our a-Si:H/SiNWs with those of amorphous silicon nanowires, it was found that the absorption edge located on the wavelength corresponds to the a-Si bandgap [31].

Lastly, broadband optical absorption combined with a low reflection value is a significant advantage of a-Si:H/SiNWs compared with a-Si thin films and silicon surfaces. This suggests that a-Si:H/SiNWs can be used as effective antireflection coating for silicon solar cells.

**Figure 4:**
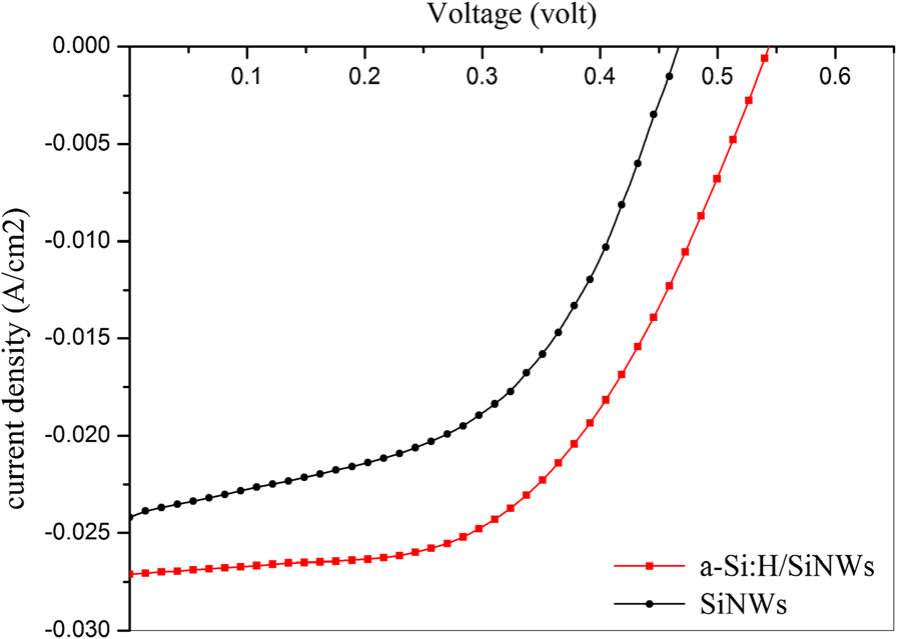
Electrical performance of a-Si:H/SiNW and SiNW solar cells.

Figure 4 and Table 1 present the electrical performance of a-Si:H/SiNW and SiNW solar cells. The values show that the a-Si:H shell highly improves surface passivation, leading to an increase of open-circuit voltage (*V*_oc_), from 481 to 553 mV, with around 15% enhancement. This passivation enhancement is related to the high content of hydrogen in the a-Si:H shell, as shown earlier in the FTIR results. Hydrogen atoms diffuse inside the SiNW core and passivate the recombination centers. Consequently, elimination of the recombination centers caused enhanced collection of electron–hole pairs leading to increased *V*_oc_ that reveals a relatively low surface recombination velocity between the SiNWs and front electrode as well good bulk properties of the SiNWs. A relative explanation for the highly increased *V*_oc_ is the assumption of Smith et al. [32] that the majority of generated carriers in the amorphous Si shell spread into the SiNW core, and then carriers are transported to the front electrode as photocurrent. The high mobility of the SiNW core leads to enhanced transportation of the carriers and finally enhanced surface passivation of the SiNW surface.

**Table 1:** Performances of the SiNW solar cells with and without a-Si:H shell

**Sample**	***V***_**oc**_	***J***_**sc**_	**FF**	**PCE**
	**(V)**	**(mA/cm**^**2**^**)**	**(%)**	**(%)**
a-Si:H/SiNWs	0.553	27.1	55.0	8.03
SiNWs	0.481	24.2	51.0	5.94

Referring to Figure 4 and Table 1, there is also clear improvement in the short-circuit current density (*J*_sc_). This increasing trend could not be mainly related to the trapping effect of the a-Si:H/SiNW core/shell structure. As mentioned previously, the reflection of the a-Si:H/SiNWs is slightly higher than that of the SiNWs alone. Subsequently, the main factor that leads to such increment in electrical performance is the low recombination velocity which becomes less due to the passivation effect of the a-Si:H shell as described earlier.

The calculated fill factor (FF) of the a-Si:H-passivated SiNW solar cell improved by 8%, reaching 55%. This improvement can be attributed to the decreasing contact area between the electrode and SiNWs. However, the original FF of the nonpassivated SiNW solar cell is still low. This low magnitude is more related to the main problem facing SiNW solar cells, i.e. electrode contact resistance. Hopefully, by solving the metal contact problem, the fill factor can be improved.

Our a-Si:H-passivated SiNW solar cell exhibits an improved efficiency by 37%, an open-circuit voltage by 15%, a short-circuit current by 12%, and a fill factor by 8%, as compared to the SiNW solar cell without a-Si:H. It is anticipated that the recombination rate and surface state density are decreased when the a-Si:H shell was used. However, more optimization of the a-Si:H shell thickness is needed. Moreover, more theoretical and experimental perceptions of the a-Si:H/SiNW interface is needed to maximize the a-Si:H passivation effect on the SiNW surface.

## Conclusions

In summary, vertically aligned Si nanowires have been synthesized and implemented to a Si nanowire/a-Si:H core/shell solar cell for photovoltaic devices. Optical studies reveal that the a-Si:H/SiNWs have low reflectivity (around 5.2%) in the entire spectral range (250 to 1,000 nm) of interest for solar cells with a superior absorption property with an average over 87% of the incident light. In investigating the passivation effect of the a-Si:H shell, we find that the combination of the a-Si:H shell and SiNW solar cell leads to enhanced power conversion efficiency, open-circuit voltage, and short-circuit current by more than 37%, 15%, and 12%, respectively, compared to the SiNW cells. This is mainly due to the suppression of the surface recombination of the large surface area of SiNWs. We expect that the a-Si:H will have a significant role in passivation of the SiNW surface with more optimization of its thickness and more theoretical understanding of its interface with SiNWs.

## Abbreviations

AgNO3: Silver nitrate; AM: Air mass; ARC: Antireflection coating; a-Si:H: Hydrogenated amorphous silicon; FESEM: Field emission scanning electron microscopy; FF: Fill factor; FTIR: Fourier transform infrared spectroscopy; H2O2: Hydrogen peroxide; H2SO4: Sulfuric acid; HF: Hydrofluoric; HIT: Heterojunction with intrinsic thin layer; Jsc: Short-circuit current; PCE: Power conversion efficiency; PECVD: Plasma-enhanced chemical vapor deposition; PSG: Phosphosilicate glass; SiNW: Si nanowire; TEM: Transmission electron microscopy; VLS: Vapor–liquid-solid; Voc: Open-circuit voltage.

## Competing interests

The authors declare that they have no competing interests.

## Authors’ contributions

ESA conceived of the study and participated in its design and coordination as well carried out the fabrication and characterization of the a-Si:H/SiNW solar cell. Moreover, ESA interpreted the results and prepared the manuscript. MYS was involved in drafting and revising the manuscript. MHR, KS, ESA, and MYS have given final approval of the manuscript to be published.
